# Expression of HLA-I, CD8, and CD4 and Their Clinical Significance in Cervical Cancer

**DOI:** 10.4021/wjon286w

**Published:** 2011-02-26

**Authors:** Jiang Tao Fan, Yan Liao, Xiao Hui Si, Xiao Li Geng, Wei Wei, Qing Li Xie

**Affiliations:** a Department of Obstetrics and Gynecology, the First Affiliated Hospital of Guangxi Medical University, Nanning 530021, China; b Department of Pathology, the First Affiliated Hospital of Guangxi Medical University, Nanning 530021, China

**Keywords:** HLA class I antigen, CD8, CD4, Cervical cancer, Cervical intraepithelial neoplasia

## Abstract

**Background:**

The local tissue immune status may play a role in the progression of cervical cancer. The aim of our study is to examine the expression of HLA-I, CD8 and CD4 in various cervical diseases and investigate their association with cervical cancer.

**Methods:**

We chose the tissues of cervical cancer, cervical intraepithelial neoplasia (CIN), chronic cervicitis and peri-cancer tissues, and then detected the expression of HLA-I, CD8 and CD4 using SP immunohistochemistry. The associations of the expression of HLA-I, CD8 and CD4 with the clinicopathologic profiles of the patients were analyzed.

**Results:**

The percentage of positive tissue staining of HLA class I antigen in cervical cancer, CIN, chronic cervicitis and peri-cancer tissues were 40%, 95%, 100.0% and 100.0%, respectively. And the percentage of CD8 in various tissues was 35%, 95%, 100% and 100.0%, respectively. The positive tissue staining percentage of CD4 in the tissues above was 45%, 80%, 100% and 100%, respectively. The percentage of positive tissue staining of HLA-I, CD8 and CD4 were significantly lower in tissues of cervical cancer when compared with other tissues (P < 0.01). No correlation between positive tissue staining of HLA-I, CD8, and CD4 and clinicopathologic profiles was observed (P > 0.05). A positive correlation was found between HLA-I and CD8 expression (Spearman’s correlation *r_s_* = 0.913, P < 0.001).

**Conclusions:**

The expression of HLA-I, CD8 and CD4 are down-regulated or deleted in CIN and cervical cancer, and they may play important roles in the development and progression of CIN and cervical cancer.

## Introduction

Cervical cancer is the second leading cause of cancer related death among women worldwide. Approximately 500,000 new cases are diagnosed each year and nearly 50% deaths are attributable to the disease, over 80% of which occur in developing countries [1]. In china, about 48,000 new cases of cervical cancer are diagnosed each year, which shows a trend of younger and younger [2]. A wealth of epidemiological, biological, pathological, virological and clinical evidence have led to the conclusion that virtually all the cases of cervical cancer, and its precursor intraepithelial lesions, are results of infection with one or other of a subset of human papillomaviruses (HPVs). In different areas of the world, HPV infection distribution in women with cervical cancer is between 86% and 94% [3]. Several lines of independent evidence support the importance of the cellular immune response in the pathogenesis of cervical cancer. More than 60% of HPV-positive, mildly dysplastic lesions resolves spontaneously, and immunodeficiency is associated with increased incidence of persistent HPV infection. Doobar’s research [4] also indicates the host’s immune system may play an important role in cervical cancer occurrence and progression. Human leukocyte antigen-I (HLA- = 1 \* ROMAN I), CD8 and CD4 molecules are important parts of human immune system. The major function of HLA-I is to present viral antigens to immune cells such as T cells then to induce the antigen-specific CTL activity in human body. The low or loss expression of such molecules probably plays an important role in the immune escape of the tumor cells. In our study, we examined the expression of HLA-I, CD8 and CD4 in tissues of cervical cancer, cervical intraepithelial neoplasia (CIN), chronic cervicitis and peri-cancer tissues using SP immunohistochemistry, to understand local immune state of cervix and investigate their association with cervical cancer.

## Materials and Methods

### Source of material

Sixty surgical specimens were collected from Department of Obstetrics and Gynecology, the First Affiliated Hospital of Guangxi Medical University between 2007 and 2009. These specimens contained 20 samples of chronic cervicitis with age between 38 and 64 (47.5 ± 5.6), 20 samples of CIN (CIN = 1 \* ROMAN I: 7, CIN = 2 \* ROMAN II: 5, CIN = 3 \* ROMAN III: 3, carcinoma in situ: 5) with age between 32 and 64 (43.1 ± 8.8), and 20 samples of cervical cancer (clinically staging by FIGO2000: = 1 \* ROMAN I - 14, = 2 \* ROMAN II - 5, = 3 \* ROMAN III - 1; G1 4, G2 9,G3 7; with lymph node metastases 4, no lymph node metastasis 16) with age between 29 and 65 (44.4 ± 9.3). There were also 20 samples of peri-cancer tissue with the same pathological symbol as cervical cancer. All of them did not accept radiotherapy, chemotherapy and immunotherapy before surgery. Each slide was revised by two senior pathologists in order to verify the diagnosis.

### Immunohistochemistry of HLA-I, CD8 and CD4

Briefly, 5µm, unstained sections from each of all 80 specimens were prepared for immunohistochemical staining. Epitope retrieval by microwave was performed. The sections were incubated with mouse monoclonal antihuman HLA-I, CD8 and CD4 antibodies (diluted 1 : 50, 1 : 30, and 1 : 30 as the working concentration, respectively). Antigen-antibody binding was demonstrated using the HRP enzyme labeled polymer conjugated to mouse secondary antibodies by the dextran-polymer technique. Sections of tonsil were used as positive controls. Negative control tissue sections were incubated with PBS instead of first antibody as blank controls.

### Result judgement and statistics

The extent of immunohistochemical staining for HLA-I, CD8 and CD4 were assessed according to the ImmunoReactive Score that evaluated the proportion of the cells expressing such molecules and the intensity of the staining. Staining intensity was graded as 0, 1, 2, 3 (0 for no dyeing noting, 1 score for flaxen, 2 scores for tan, 3 scores for brown); percentage of positive cells examined was scored as 0 (negative), 1(< 10%), 2 (11% - 25%), 3 (26% - 50%), and 4 (> 50%). The two scores were multiplied and the ImmunoReactive Score was determined as follows: 0 to 2 as negative (-); 3 to 4 as weak (+); 5 to 8 as positive (++); 9 to 12 as strong positive (+++).

The comparisons of HLA-I, CD8 and CD4 in each group were done using the chi-square test. The correlation between HLA-I and CD8 was investigated using Spearman rank correlation analysis. SPSS statistical software (version 16, SPSS Inc., Chicago, IL) was used. A P < 0.05 was considered significant.

## Results

### Expression of HLA-I in each group

**Figure 1:**
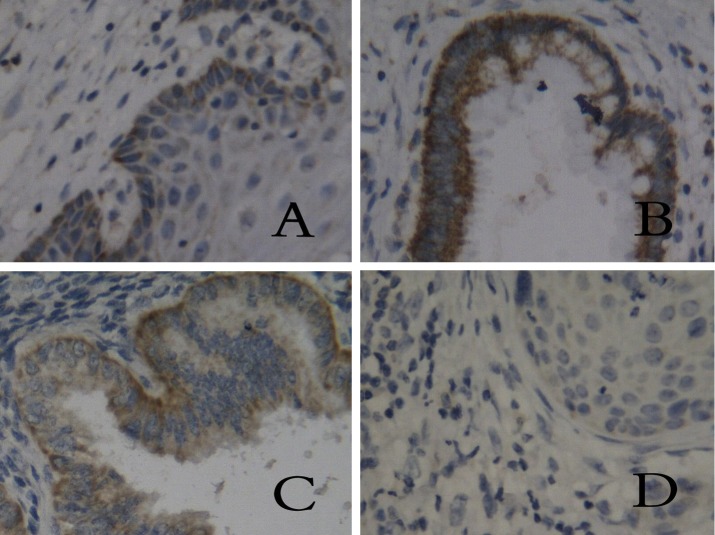
Tissue staining of HLA-I in different groups. (A) chronic cervicitis, (B) peri-cancer tissue, (C) carcinoma in situ, (D) cervical cancer. (SP, × 400)

HLA-I expression was localized on the membrane of all nuclear cells, dyeing from flaxen to brown, as well as in cytoplasm. In the chronic cervicitis group, the percentage of positive tissue staining was 100% (20/20), including 8 cases (+), 9 cases (++) and 3 cases (+++); while in CIN group, the percentage was 95% (19/20), including 1 case (-), 11 cases (+) and 8 cases (++). In cervical cancer group, the percentage of positive tissue staining was 40% (8/20), including 12 cases (-), 7 cases (+), and 1 case (++); while the percentage of positive tissue staining in peri-cancer group was 100% (20/20), including 7 cases (+), 11 cases (++) and 2 cases (+++). The percentage of positive tissue staining of HLA-I was significantly lower in cervical cancer group than in CIN, chronic cervicitis and peri-cancer group (P < 0.01) (Fig. 1).

No statistical different positive tissue staining of HLA-I was found in cervical cancer group when correlated with clinical stages, cell differentiation, and lymph node metastasis. No statistical difference of HLA-I tissue staining was found in each CIN group (P > 0.05).

### Expression of CD8 and CD4 in different groups

**Figure 2:**
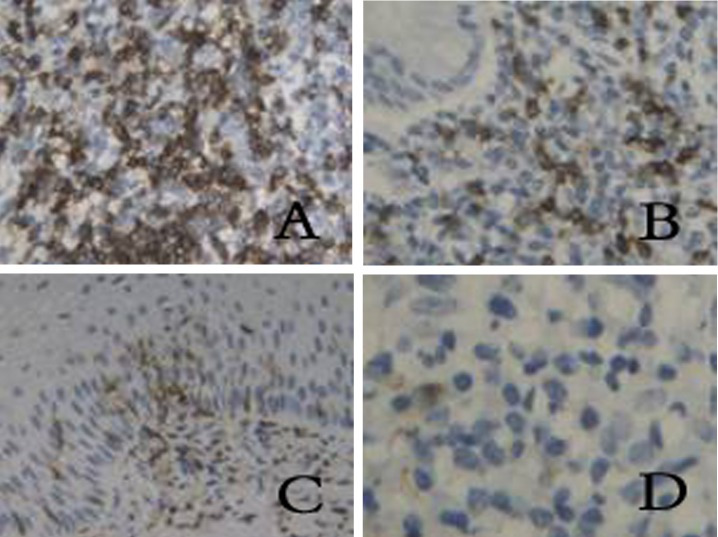
Expression of CD8 in different groups. (A) chronic cervicitis, (B) peri-cancer tissue, (C) CIN, (D) cervical cancer. (SP, × 400)

**Figure 3:**
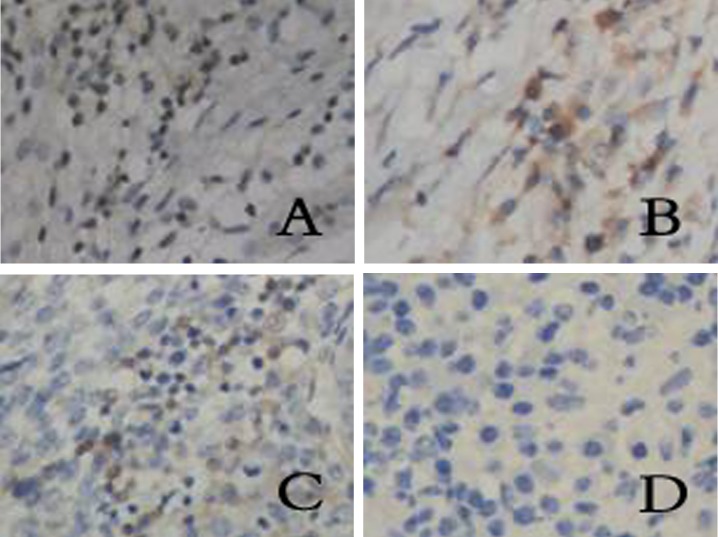
Expression of CD4 in different groups. (A) chronic cervicitis, (B) peri-cancer tissue, (C) CIN, (D) cervical cancer. (SP, × 400)

CD8 and CD4 molecules dyeing from flaxen to brown were localized on membrane or in cytoplasm of lymphocytes, which distributed in stroma of epithelium and stratum basale, as well as a few expressions in cervical cancer tissues. The percentage of positive tissue staining of CD8 in chronic cervicitis group was 100% (20/20), including 8 cases (+), 11 cases (++), and 1 case (+++). The percentage of positive staining in CIN group was 95% (19/20), including 1 case (-), 12 cases (+), and 7 cases (++). But in cervical cancer group, the positive expression rate decreased to 35% (7/20), including 13 cases (-) and 7 cases (+). The percentage of positive tissue staining of CD8 in peri-cancer group was 100% (20/20), including 8 cases (+), 10 cases (++), and 2 cases (+++). The percentage of positive tissue staining of CD4 in chronic cervicitis group, CIN group, cervical cancer group, and peri-cancer group was 100% (20/20), 80% (16/20), 45% (9/20), and 100%(20/20), respectively. Results indicated that expression rates of CD8 and CD4 molecules in cervical cancer group were significantly lower than in CIN, chronic cervicitis and peri-cancer group (P < 0.01) (Fig. 2, 3). Tissue staining of HLA-I, CD4 and CD8 in cervical cancer group is showed in Table 1.

**Table 1:** Expression of HLA-I Antigen, CD8 and CD4 in Cervical Cancer

Index	Case	Expression of HLA-I antigen				Expression of CD8				Expression of CD4			
		-	+	++	+++	-	+	++	+++	-	+	++	+++
Clinicopathological stage													
Stage I	14	8	5	1	0	9	5	0	0	8	5	1	0
Stage = 2 \* ROMAN II	6	4	2	0	0	4	2	0	0	3	1	2	0
Differentiation													
High	4	3	1	0	0	3	1	0	0	3	0	1	0
Moderate	9	5	3	1	0	6	3	0	0	5	3	1	0
Low	7	4	3	0	0	4	3	0	0	3	3	1	0
Lymph node metastassis													
No	16	11	5	0	0	10	6	0	0	8	5	3	0
Yes	4	3	1	0	0	3	1	0	0	3	1	0	0

No statistical different positive tissue staining of CD8 and CD4 was found in cervical cancer group when correlated with clinical stages, cell differentiation, and lymph node metastasis. No statistical difference of CD4 and CD8 tissue staining was found in each CIN group (P > 0.05).

### The tissue staining of HLA-I and CD8 in different groups

Table 2 shows the correlation between tissue staining of HLA-I and CD8 in each groups. The percentage of negative or (+) tissue staining of HLA-I in all group is 65% (39/60), while the percentage of CD8 is 68.3% (41/60). With the decrease of positive or strong positive tissue staining of HLA-I in cervicitis, CIN and cervical cancer groups, the positive tissue staining of CD8 decreases. There is a positive correlation between the two molecules (r_s_ = 0.913, P = 0.000).

**Table 2:** Correlations Between HLA-I and CD8 in Different Groups

		HLA-I				
		+	++	+++	-	total
	**+**	25	2	0	0	27
	**++**	0	16	2	0	18
**CD8**	**+++**	0	0	1	0	1
	**-**	1	0	0	13	14
	**total**	26	18	3	13	60

## Discussion

Cervical cancer and precancerous cervical lesions constitute a major problem in women’s health. A substantial number of epidemiological studies have been performed, which point to high-risk HPV as the primary risk factor for cervical cancer. It is well established that persisting HPV infections were the most significant risk factor for cervical cancer. Virtually all cervical cancers contain the genes of high-risk HPVs, most common types 16, 18, 31, and 45. Numerous lines of evidence suggest that cell-mediated immune responses are important in controlling established HPV infections as well as HPV-associated neoplasms, especially in clearance of tumor cells [5]: (a) the prevalence of HPV-related diseases (infections and neoplasms) is higher in patients with impaired cell-mediated immunity, including transplant recipients and HIV-infected patients; (b) infiltrating CD4^+^ and CD8^+^ cells have been observed in spontaneously regressing warts. As an important component of the immune system, HLA system is able to present antigenic peptide to antigen-specific T cells, and then trigger the immune response to eliminate tumor specific proteins. The main function of HLA-I is to combine with processed endogenous antigen, forming HLA-antigen complex and presenting them to the surface of cell. CD8^+^ T cells can identify the complex, then be activated and differentiate into cytotoxic T cells (Tc or CTL), which can directly kill target cells via secreting substances such as perforin [6], or inducing target cell apoptosis through Fas/FasL way to result in special lethality for target cells [7]. So any modification of the celluar antigen-presenting system can correlate with the escape of CIN and carcinoma in situ from immunological control then progress to invasive carcinoma. The escape from immune-surveillance mechanisms emerges as one important step in the progression of HPV-linked tumors. Scholars from different countries have found that HLA-I was expressed weakly or even not expressed at all in the cancer tissue of breast, colon, ovary and cervix, and so on [8, 9]. Low HLA-I expression could not present antigenic peptides to the CTL cells. Then the tumor cells can avoid the specific killing effects of CTL cells, and then continue to progress and metastasize. As to CD4^+^ cells, a kind of T lymphocyte surface glycoprotein, can combine with HLA-II molecules, which are mainly expressed on the surface of professional antigen presenting cells (APC), and be activated into helper T cells to identify exogenous antigen and start the immune response and regulate the immune function after the combination [10]. The low expression of CD4 molecules can weaken the immune activity fighting against cancer cells.

Our immunohistochemical study demonstrated that HLA-I antigen was expressed in all cases in chronic cervicitis group and CIN group. The percentage of positive tissue staining was statistically low in cervical cancer group. The expression of CD8^+^ T cells and CD4^+^ T cells were also statistically low in the cervical cancer group than in other groups. The low expression of HLA-I and CD8^+^ T cells in cervical cancer tissue may indicate that tumor cells exhibit HLA-I downregulation, with a resultant loss of antigen presentation capability and downregulation of endogenous peptide processing machinery for MHC presentation. The significance of this is that cells expressing high levels of tumor-specific proteins may fail to be recognized and destroyed by CTL. This kind of escape from surveillance of local immune system can lead to the progression and metastasis of tumor cells. Yue Qi’s research has shown the similar results [9]. Data from other researchers demonstrated that local immune status might be a critical factor in fighting against HPV-associated neoplasia [11, 12].

In conclusion, the local expression of HLA-I, CD8 and CD4 in cervical tissue may be involved in the occurrence and progression of cervical carcinoma. Immune therapy is now becoming a new treatment alternative following surgery, radiotherapy and chemotherapy for malignant tumors. HLA, CD8 and CD4 turn into the new consideration of immune therapy. Options to enhance the local or systemic immune status can reduce the sneaking through of the tumor cells from the immune surveillance.
